# Evolutionary history of mental glands in turtles reveals a single origin in an aquatic ancestor and recurrent losses independent of macrohabitat

**DOI:** 10.1038/s41598-021-89520-w

**Published:** 2021-05-17

**Authors:** Alejandro Ibáñez, Uwe Fritz, Markus Auer, Albert Martínez-Silvestre, Peter Praschag, Emilia Załugowicz, Dagmara Podkowa, Maciej Pabijan

**Affiliations:** 1 Department of Comparative Anatomy, Institute of Zoology and Biomedical Research, Jagiellonian University, 30-387 Kraków, Poland; 2 Department of Ecology and Vertebrate Zoology, Faculty of Biology and Environmental Protection, University of Łódź, 90-237 Łódź, Poland; 3 Museum of Zoology, Senckenberg Dresden, 01109 Dresden, Germany; 4 Catalonian Reptile and Amphibian Rehabilitation Center (CRARC), 08783 Masquefa, Spain; 5 Turtle Island, 8054 Graz, Austria

**Keywords:** Macroecology, Evolution, Zoology

## Abstract

Despite the relevance of chemical communication in vertebrates, comparative examinations of macroevolutionary trends in chemical signaling systems are scarce. Many turtle and tortoise species are reliant on chemical signals to communicate in aquatic and terrestrial macrohabitats, and many of these species possess specialized integumentary organs, termed mental glands (MGs), involved in the production of chemosignals. We inferred the evolutionary history of MGs and tested the impact of macrohabitat on their evolution. Inference of ancestral states along a time-calibrated phylogeny revealed a single origin in the ancestor of the subclade Testudinoidea. Thus, MGs represent homologous structures in all descending lineages. We also inferred multiple independent losses of MGs in both terrestrial and aquatic clades. Although MGs first appeared in an aquatic turtle (the testudinoid ancestor), macrohabitat seems to have had little effect on MG presence or absence in descendants. Instead, we find clade-specific evolutionary trends, with some clades showing increased gland size and morphological complexity, whereas others exhibiting reduction or MG loss. In sister clades inhabiting similar ecological niches, contrasting patterns (loss vs. maintenance) may occur. We conclude that the multiple losses of MGs in turtle clades have not been influenced by macrohabitat and that other factors have affected MG evolution.

## Introduction

The vast diversity of signals used in animal communication stems from evolutionary processes such as natural and sexual selection that either promote or constrain signaling in specific situations^1,2^. For instance, ornamental colors reliably signaling for individual condition may be favored by sexual selection by increasing reproductive success^3–5^. However, natural selection (e.g. predator pressure) may constrain the development of striking coloration due to the higher detectability of showy individuals to predators^6,7^. Other factors, such as habitat conditions, may also influence intraspecific signaling^8–10^, adding a further dimension of complexity.

In animals, pheromones or chemical signals are important in social contexts. Usually an individual produces and releases a chemical substance (e.g. pheromone) to the environment that provokes a physiological and/or behavioral reaction in the receiver^11^. So far, most research on chemical communication has been done in insects, for which the chemical nature of specific molecules and their mechanism of action is known to some extent^12,13^. Although many vertebrates are adept at detecting odoriferous stimuli, much less research has been done in this group compared to insects^13^. Recent decades have seen advances in some groups of vertebrates, e.g. mammals^14,15^, amphibians^16–18^ and squamates^19,20^. However, studies focusing on other vertebrates such as birds and turtles remain scarce^21^. Indeed, birds were traditionally thought to have a poor sense of smell but have lately been demonstrated to use chemical signals to communicate in social contexts such as partner discrimination^22^ and even kin recognition^23,24^.

Environmental conditions may impose constraints on pheromone transmission and detectability and may ultimately affect chemical signal design^8,25,26^. The use of chemosignals may be more suitable in certain habitats than others. For instance, many species of lizards possess femoral glands—organs involved in the production of chemosignals—that are more (or less) developed, depending on the environment. Shrub-climbing lacertid lizards have a lower number of femoral gland pores than lizards living on sandy, rocky or vegetated substrates, which can be interpreted as a smaller investment in chemical signaling^27^. Similarly, in lizards of the genus *Sceloporus*, arboreal species have lower numbers of pores than terrestrial ones^28^.

Another factor influencing the evolution of chemical communication is the effect of relatedness among species, i.e. the tendency for lineages sharing recent ancestors to exhibit greater trait similarity than phylogenetically divergent taxa. For instance, in lizards, phylogenetic relatedness was found to be a more powerful predictor of number of organs involved in chemical communication than adaptation to similar environment^27,29^.

Turtles and tortoises (i.e. chelonians) possess a large repertory of olfactory receptor genes that is comparable to, or even higher than, that of mammals^30^. Both olfactory and vomeronasal senses are well developed in turtles^31^ and the relative importance of either of these may be determined by the extent of aquatic habitat use^32^. In particular, the proportion of functional olfactory receptor genes was shown to be lower in aquatic turtles than in terrestrial species, suggesting that volatile olfactory cues are less important in water than on land^32^. Schwenk^31^ suggested that in non-avian reptiles, the vomeronasal system alone functions during aquatic foraging, while the olfactory system is reserved for aerial olfaction.

**Figure 1:**
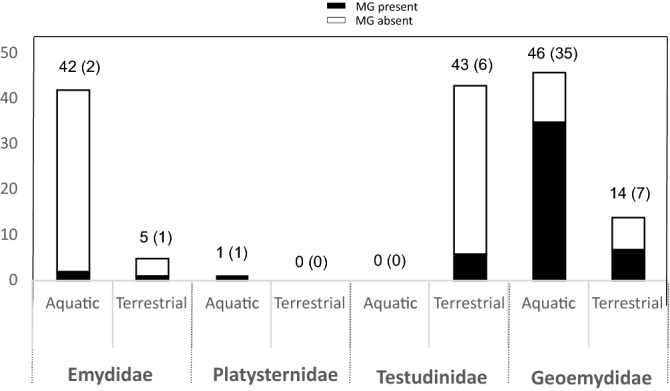
Summary of the number of species with and without mental glands (MGs) by family and habitat (based on the species included in the phylogenetic timetree of Pereira et al.^43^—i.e. 292 species). Total number of species in a family and habitat is shown at the top of the bar. The value in brackets is the number of species with MGs in a given category (family/habitat).

Chemical compounds potentially involved in intraspecific communication in turtles are produced by mental or chin glands, Rathke glands and cloacae^33–36^. Mental glands (MGs) are paired structures of epidermal origin situated laterally behind the tip of the mandible in the skin of the chin, producing holocrine secretions^37–39^. Behavioral studies and observations on tortoises of the genus *Gopherus* have indicated that MG secretions play an important role in mate choice and/or conspecific discrimination^40–42^. Winokur and Legler^39^ assessed the status of MGs in all families of extant turtles and provided a histological description for several species. These authors found MGs in emydid, platysternid, testudinid and geoemydid (formerly batagurid) turtles and suggested that these structures are homologous based on the position of the gland, anatomical/histological similarity and continuous variation of MG expression across species^39^. However, this preliminary hypothesis lacked a phylogenetic framework. Here, we formally test whether MGs are homologous or if they evolved repeatedly along the turtle phylogeny. For this first aim, we used the existing data^39^ together with our own (re-)assessment of MG presence/absence in many turtle species, including key taxa that were not examined previously, compiling an extensive dataset on MG occurrence. Based on a comprehensive and time-calibrated turtle phylogeny^43^, we infer the evolutionary history of this organ. The second aim of this study was to test for an association between gland status and general macrohabitat (aquatic vs. terrestrial). Our dataset indicates that most (~ 73%) of the 52 species that have MGs are aquatic, while the rest inhabit terrestrial environments (~ 27%; see Results and Fig. 1). We hypothesize a close link between the presence of MGs and freshwater environments. Chemical cues could be especially useful in some wetland or riverine environments in which visibility is limited due to turbidity, curbing for example, foraging efficiency^44^. In lentic water bodies, chemosignals may persist for relatively long periods of time, and thus may be particularly effective for intraspecific communication. However, chemical signals could fade out rapidly due to wind or heat in terrestrial environments^8^. Given that most of the terrestrial chelonians (e.g. Testudinidae) inhabit hot or semiarid habitats^45^, one hypothesis is that the high temperatures of these environments could compromise the persistence of chemical signals, prompting the loss of MGs for most species living on land. Using a comparative macroecological approach, we address whether the evolution of MGs has been influenced by macrohabitat.

## Results

### Mental gland distribution and structure

**Figure 2:**
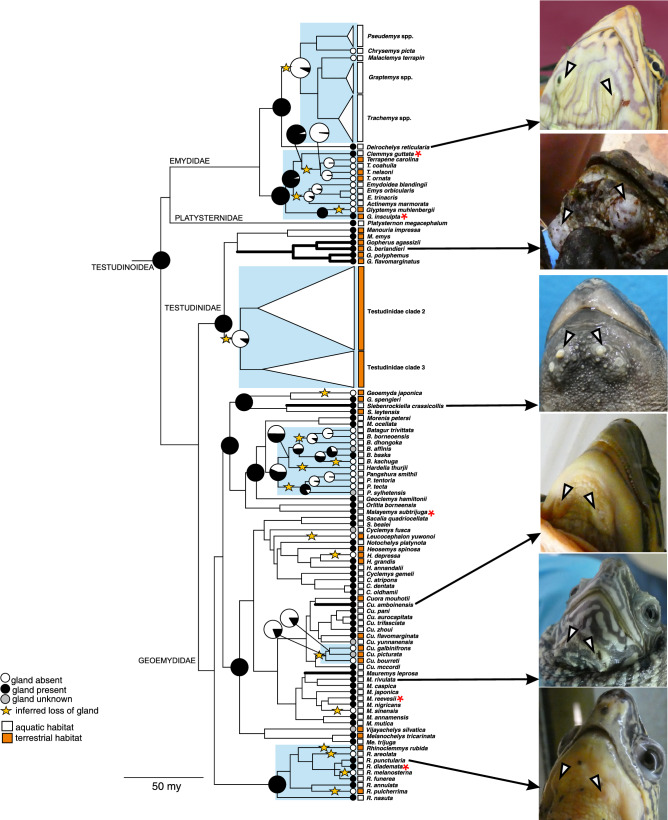
Maximum likelihood inference of ancestral states in mental gland (MG) status in extant Testudinoidea based on the molecular timetree of Pereira et al.^43^, allowing for different transition rates between states (ARD model). Pie charts show marginal likelihood estimates for states at ancestral nodes; note that those showing full support for one of the states were mostly omitted except for some key ancestral nodes such as the mrca of Testudinoidea. Stars indicate an inferred loss of MGs, based on one interpretation of the historical scenario. Lineages with the largest MGs are emphasized by thick black branches. Blue shading indicate clades in which MGs have undergone a reduction or were lost in multiple species. Species with very reduced and likely functionless forms of MGs (“vestigial”) are marked with an asterisk (only species with at least four males assessed macroscopically are highlighted, in the case of *Malayemys subtrijuga* MGs were only found in females). Circles and squares at tips show mental gland status and macrohabitat, respectively, for extant species. Photographs on the right provide illustrations of mental gland phenotypes in representatives of major clades of Testudinoidea (arrows point to MGs). Species names at the terminal labels as in the original publication^43^, inclusive of putative misidentifications.

Taking into account our present and previous surveys^39^, more than 1700 specimens were checked for MG absence or presence (Table 1). In further analysis, our dataset was pruned to include the 292 species present in the Pereira et al.^43^ timetree (see below). A total of 234 out of 292 turtle species included in our analysis were scored as lacking MGs. Mental glands were present in 52 species from the families Geoemydidae (42 species), Emydidae (3 species), Testudinidae (6 species) and Platysternidae (1 species). Mental gland status was ascribed as unknown in a total of 6 species, all belonging to the Geoemydidae family. Macroscopic inspection of MGs showed that these were large and prominent in only a few species, while they were relatively small or even extremely reduced (likely vestigial) in others (Fig. 2; Figs. S1–S2 for a comparison on the macroscopic aspect among several taxonomic groups). In addition, MGs were polymorphic and appeared in a variable number of specimens depending on the species (Suppl. Table S1).

**Table 1:** Results of surveys for mental gland (MG) status for all extant turtle families from Winokur and Legler^39^ and this study (in brackets).

Winokur and Legler^39^ (our study)	Specimens MGs	Total specimens
Family Chelydridae	0 (–)	29 (0)
Family Kinosternidae	0 (0)	210 (4)
Family Dermatemydidae	0 (–)	15 (0)
Family Trionychidae	0 (–)	27 (0)
Family Carettochelyidae	0 (–)	6 (0)
Family Cheloniidae	0 (–)	50 (0)
Family Dermochelyidae	0 (–)	5 (0)
Family Pelomedusidae	0 (–)	11 (0)
Family Podocnemididae	0 (–)	13 (0)
Family Chelidae	0 (–)	52 (0)
**Family Platysternidae**	**8 (9)**	**12 (13)**
**Family Testudinidae**	**22 (19)**	**108 (64)**
**Family Emydidae**	**16 (11)**	**140 (101)**
**Family Geoemydidae**	**127 (285)**	**319 (560)**

Families in which MGs were found are highlighted in bold. All examined specimens from both studies are listed.

**Figure 3:**
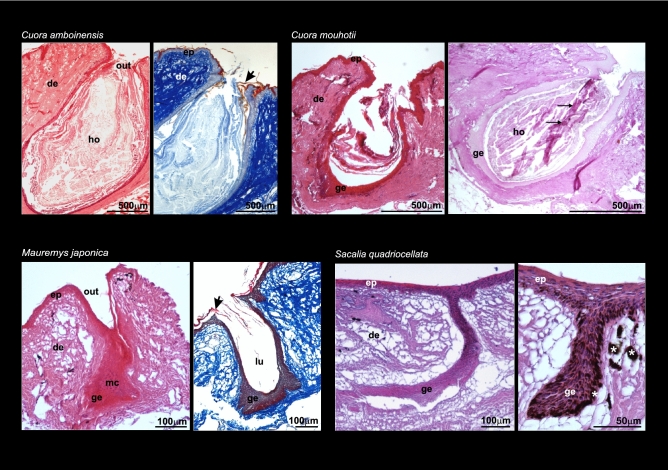
Histological plate showing mental gland structure in males of four species of geoemydid turtles. Staining techniques: *Cuora amboinensis* and *Mauremys japonica*; Haematoxylin Eosin (left) and Mallory’s Trichrome (right). *Cuora mouhotii*; Haematoxylin Eosin (left) and Periodic Acid-Schiff stain (right). *Sacalia quadriocellata*, Haematoxylin Eosin. Arrows point to keratinized layer (red colored) in *Cuora amboinensis* and *Mauremys japonica*. Arrows in *Cuora mouhotii* point to holocrine secretion reacting positively to PAS. In *Sacalia quadriocellata*, asterisks point to melanocytes. Abbreviations: de, dermis; ep, epidermis; ge, glandular epithelium; ho, holocrine secretion; lu, lumen; mc, mature cell; out, outlet of the gland.

**Figure 4:**
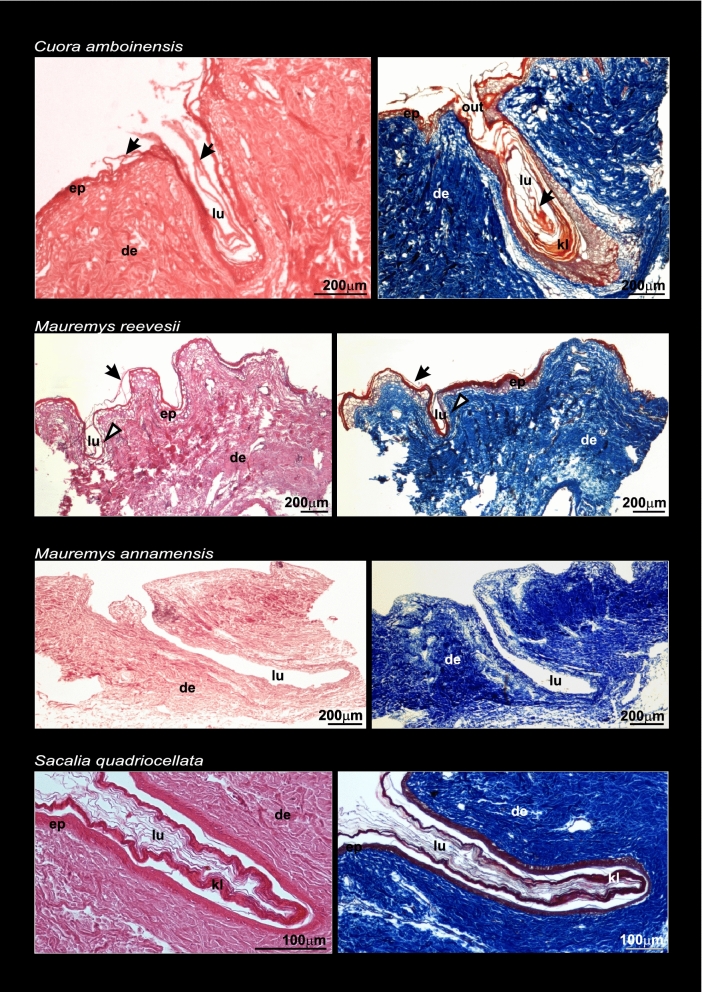
Histological plate showing mental gland structure in females of four species of geoemydid turtles. Staining techniques are Haematoxylin Eosin (left) and Mallory’s Trichrome (right). Arrows point to the keratinized layer (red color). Arrowheads point to a very small invagination in *Mauremys reevesii* (a likely vestigial form of MGs). In *M. annamensis,* the tissue shows signs of degradation such as the lack of a keratinized layer. Abbreviations: de, dermis; ep, epidermis; kl: keratinized layer; lu, lumen; out, outlet of the gland.

Histological examination of six geoemydid species showed that MGs are epidermal invaginations through the dermis with different levels of complexity and shape varying among species and sexes (Figs. 3 and 4). The highest degree of development was shown by males of *Cuora amboinensis* and *C. mouhotii* (Fig. 3). In these species MGs are sac-shaped, and holocrine secretion accumulating in the lumen of the gland is visible. In *C. mouhotii* the glandular secretion reacts positively to Periodic Acid-Schiff (PAS) stain, indicating the presence of polysaccharides. Male specimens of *Sacalia quadriocellata* and *Mauremys japonica* had relatively smaller and simpler MGs than *Cuora* but still showed well-developed glands, and the presence of thick glandular tissue indicated these are potentially active (Fig. 3). In *S. quadriocellata*, despite that the lumen of the gland was not visible, a thick glandular epithelium was present. There were no signs of a keratinized layer in the middle part of the gland as in other holocrine glands (Fig. 3). These differences in the complexity of MGs could be at least in part due to the breeding cycle of the individuals—it is possible that both specimens of *Cuora* (*C. amboinensis* and *C. mouhotii*) were reproductively active, while the other two specimens (*S. quadriocellata* and *M. japonica*) were outside of the reproductive season. In the case of females, MGs consisted of heavily keratinized invaginations through the dermis and were relatively simple and smaller compared to male glands (Fig. 4). In addition, holocrine secretion was not found in female MGs, indicating quiescence. A comparison of MG structure in both sexes for *C. amboinensis* and *S. quadriocellata* clearly showed that MGs in females are reduced and lack glandular epithelium (producing holocrine secretion) that contrasts with the better-developed glands in the males of these species. A specimen of *Mauremys annamensis* sexed as a female showed a relatively deep but narrow MG. In the case of *M. reevesii*, extremely small invaginations were detected in one of the specimens sexed as a female; histological examination showed that this is likely a vestigial form of the gland (Fig. 4). A comparison with another specimen of *M. reevesii* in which MGs were not recognized is provided in the Supplementary Material (Fig. S3). Some signs of degradation were observed in MG tissues in the histological preparations, likely due to the time elapsed between death and fixation in ethanol.

More details on the macroscopic aspect and histology of MGs as well as variation within and across species is given in the Supplementary Material (see sections *Controversial taxa and intraspecific variation; Details on the structure and evolution of MGs in turtles* and Figs. S1–S3).

### Inference of ancestral states

Our binary state reconstruction using the Pereira et al.^43^ timetree revealed that a single acquisition of mental glands in the ancestor of Testudinoidea was the most likely scenario (Table 2, Table S2, Fig. 2; see Fig. S4 for the tree including all chelonian taxa): The top three models (within 0.6 AICc units of each other) gave qualitatively similar reconstructions. Out of these, the ARD model was the least parametrized. Notably, two of the best-fitting models incorporated hidden Markov processes with a matrix category transition at the most recent common ancestor (mrca) of Testudinoidea, consistent with the ARD model. In keeping with the inferred single origin of MGs, all three models showed that the rate of losing a gland was much higher than gaining one (Table S2). In contrast, the IO model, which enforced independent origins of MGs, suggested at least 5 independent gains of MGs but was unsupported (ΔAICc = 8.8).

**Table 2:** The fit of alternative models of mental gland (MG) evolution in turtles, based on Akaike scores and weights derived from the log likelihoods of the models.

Model	− ln *L*	AICc	ΔAICc	*w*_*i*_	*N* par	*N* origins
**Mental Glands**						
ER/ARD	− 56.5	123.2	0	0.323	5	1
ARD/ARD	− 55.5	123.3	0	0.316	6	1
ARD	− 59.9	123.8	0.6	0.239	2	1
ER/ER	− 58.5	125.2	2.0	0.118	4	≥ 4
IO	− 64.0	132.1	8.9	0.004	2	≥ 5
ER	− 72.9	147.8	24.6	0	1	≥ 5
**Macrohabitat**						
ER/ER	− 52.3	112.8	0	0.622	4	≥ 12
ER/ARD	− 52.3	114.9	2.1	0.221	5	≥ 12
ARD/ARD	− 51.6	115.6	2.8	0.156	6	≥ 12
ARD	− 66.8	137.7	24.9	0	2	1
ER	− 73.2	148.5	35.7	0	1	≥ 12

*N* par. indicates the number of estimated transition parameters. *N* origins refers to the number of independent origins of MGs or terrestriality inferred by the models. ER—all transition rates equal; ARD—distinct rates for all transitions between states (“all rates different”); IO—enforced independent origins of MGs in Testudinoidea; ER/ER—model using the generalized hidden Markov process, consisting of two ER transition matrices; ARD/ARD—hidden Markov model with two ARD transition matrices; ER/ARD—hidden Markov model with one ARD and one ER transition matrices.

Under the ARD model, independent losses of MGs within Testudinoidea occurred multiple (up to 15–18) times, with at least four losses in the ancestors of most extant emydids and at least one loss in testudinids. The history of this trait in geoemydid turtles was particularly variable with reductions or losses occurring in ancestral lineages of *Batagur* + *Pangshura* + *Hardella*, as well as in at least one lineage of *Cuora*, and along the branches of species such as *Geoemyda japonica*, *Heosemys depressa*, *Leucocephalon yuwonoi* and *Mauremys sinensis*. At least four different species of *Rhinoclemmys* have lost MGs, however, we note that the remaining members of this genus all have inconspicuous glands. The status of MGs in *Batagur baska*, a highly endangered and rare species, is ambiguous and influences the total (minimum) number of independent losses in turtles: 18 or 15, depending on whether MGs in *B. baska* are present or absent. In addition, the presence of glands in *B. affinis*—the sister species of *B. baska*—is unknown, adding more ambiguity to the status of MGs for these two species (see *Controversial taxa and intraspecific variation* in the Supplementary Material for details on this issue).

We also analyzed the data using the molecular timetree of Thomson et al.^46^, published while our study was under review, and obtained similar results: A single origin of MG’s in the mrca of Testudinoidea, a consistent number and placement of MG losses, and a nearly identical ranking of models (Table S3 and Suppl. Fig. S5). The only difference was an inferred independent origin of MGs in a single geoemydid species, *Batagur baska*, in all but the two highest-ranking models. However, due to the ambiguous status of MGs in this species (see above), we consider this result highly unlikely. The two best-fitting models (ARD/ARD and ER/ARD) could not reliably distinguish between an ancestral or independent origin of MG presence in this species (Fig. S5).

For macrohabitat, the best-fitting model suggested a plesiomorphic aquatic state at the mrca of Testudinoidea (although with considerable uncertainty) and possibly at least 12 independent origins of terrestriality within this clade (Fig. S4, Table S4). Analysis with the alternative timetree^46^ gave qualitatively identical results (Table S3).

### Is mental gland evolution correlated with habitat?

**Figure 5:**
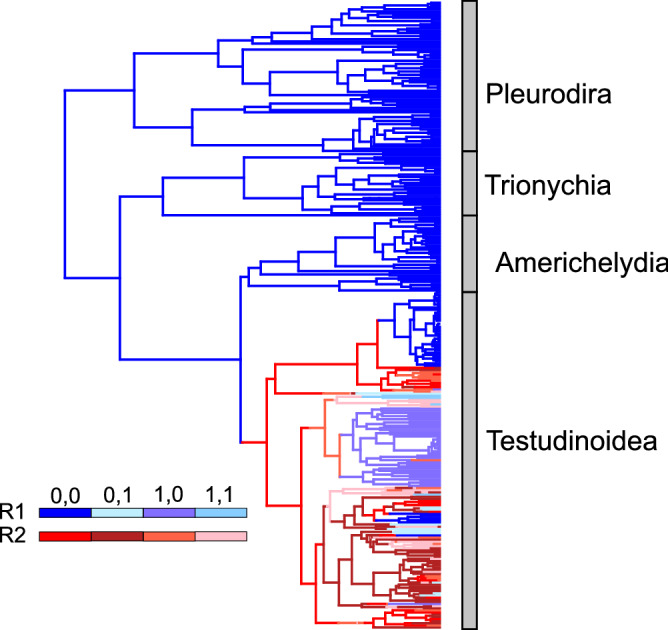
Character history for macrohabitat (aquatic/terrestrial) and mental gland occurrence (present/absent) in chelonians according to a generalized hidden Markov process with two equal rate (ER) transition matrices (ER/ER model). Branches colored in different shades of blue and red represent state combinations of the two traits evolving according to two different transition matrices (R1, R2, shown in the legend). The tree shows that the underlying evolutionary process in Testudinoidea is distinct (evolves along a different transition matrix) in relation to most other clades of turtles and tortoises. 0,0—aquatic, gland absent; 0,1—aquatic, gland present; 1,0—terrestrial, gland absent; 1,1—terrestrial, gland present.

Based on the 292 species in the timetree of Pereira et al.^43^, most turtles with MGs are aquatic (38 species, ~ 73%), while 14 species (~ 27%) live in terrestrial environments (Fig. 1). We found that generalized hidden Markov models with two transition matrices best explained the evolutionary processes producing the trait states at the terminals (Table 3, Table S5). The best model, ER/ER, was also one of the least-parameterized ones. When plotted along the branches of the turtle phylogeny, the models with the best fit invariably recovered distinct evolutionary processes occurring in the Testudinoidea clade vs. all other turtle clades (Fig. 5). A separate analysis involving only Testudinoidea (Table 3, Table S6) showed that the two ARD models best fit the data (within 2 AICc units of each other), with the IND model only marginally worse. Importantly, this analysis showed that the loss of a gland is independent of macrohabitat because the ARD model was only slightly inferior to the ARDloss model (ΔAICc = 0.9). Implementation of the Thomson et al.^46^ molecular chronogram produced congruent model rankings (Table S7).

**Table 3:** The fit of alternative models of the joint evolution of mental glands (absent/present) and macrohabitat (aquatic/terrestrial) in all chelonians and in Testudinoidea, based on Akaike scores and weights derived from the log–likelihoods of the models.

Model	− ln *L*	AICc	ΔAICc	*w*_*i*_	*N* par
**Chelonians**					
ER/ER	− 106.8	221.7	0	0.979	4
ER/ARD	− 104.3	229.7	8	0.021	10
ARD	− 110.6	237.7	16	0	8
ARDloss	− 113.1	240.5	18.8	0	7
IND	− 126.7	261.5	39.8	0	4
ER	− 146.1	294.2	72.5	0	1
**Testudinoidea**					
ARDloss	− 95.5	203.9	0	0.507	7
ARD	− 93.9	204.8	0.9	0.325	8
IND	− 98.9	206.1	2.2	0.166	4
ER	− 106.7	215.5	11.6	0.002	1

*N* par. indicates the number of estimated transition rates. ARD—distinct rates for all transitions between states (“all rates different”); ARDloss—ARD model with Q1 = Q6; IND—independent evolution between traits; ER—all transition rates equal; ER/ER—a model using the generalized hidden Markov process, consisting of two ER transition matrices; ER/ARD—a model using the generalized hidden Markov process with ARD and ER transition matrices.

## Discussion

### Evolutionary history of mental glands in turtles

Our comprehensive survey of the occurrence of mental glands (MGs) combined with a well-supported phylogeny provided a means to understand the evolutionary history of an organ involved in communication in turtles. The most likely reconstruction indicates that MGs originated once, in the ancestor of Testudinoidea, and are therefore homologous in all descendent lineages. This result is supported by broad similarity in MG position, anatomy and histology across species, although some taxa possess larger and more elaborate organs, whereas in others MGs are reduced to small and possibly nonfunctional slits in the skin of the throat^39^ (this study; see also Supplementary Material). MGs seem to have been secondarily lost multiple times within the Testudinoidea in three of four families (with the exception of the monotypic Platysternidae). Our inference of a single origin of MGs and subsequent losses in different Testudinoidea families is robust to topological differences between two recent molecular phylogenetic hypotheses for chelonians^43,46^.

Our results show that MG evolution in turtles is highly idiosyncratic, with sister clades showing different trajectories, for instance MG retention vs. MG loss in *Gopherus* + *Manouria* vs. other testudinids, respectively. The evolution of MGs seems to be particularly variable in Geoemydidae, with cases of gland loss and full functionality even within the same genera (*Cuora*, *Mauremys*). Given the generally deep divergences among branches of the turtle tree even within families, we suspect there has been ample time for individual lineages to accrue large amounts of phenotypic change in MGs. In consequence, the historical component of MG expression could have been erased in many ancient turtle clades. However, the phylogenetic effect persists in descendants of lineages that have lost MGs, as we did not infer any paths indicating that MGs could re-evolve after disappearing.

We found that generalized hidden Markov models (HMM) better approximate the complexity of MG and macrohabitat evolution in turtles. Turtles make up a relatively small but globally distributed vertebrate clade with divergences between extant families reaching the Upper Mesozoic and early Paleogene^43,46–49^. Trait evolution across such large expanses of biodiversity time may be better approximated by models that can accommodate heterogeneous rates among clades^50^. This seems to be the case for both traits that we analyzed: Nearly all highest-scoring models were HMMs with two categories of transition matrices that fit distinct parts of the turtle tree. Our analyses infer shifts toward accelerated rates of change at the origin of Testudinoidea (Fig. 5), which is not surprising given that MG presence and terrestriality seem to have arisen within this lineage. All other turtle clades reveal extremely slow rates of evolution for both traits, leading to a lack of or only a few changes in state.

### Habitat and the evolution of mental glands

Our results suggest that a plausible scenario involves the origin of MGs in an aquatic ancestor placed at the base of Testudinoidea, which is in line with the ecological settings previously determined for this ancestor as well as for crown group turtles^51^. This suggests that MGs would have evolved primarily to secrete compounds in aquatic habitats. Despite multiple shifts to a (semi)terrestrial environment within Testudinoidea, we did not detect a clear association between MG loss and terrestrial habitat within this clade, and this result was not sensitive to topological differences between the two phylogenetic hypotheses that we used in ancestral state inference. The secondary loss of MGs may have occurred at least 15–18 times according to our interpretation of ancestral states (Fig. 2), in both terrestrial and aquatic lineages. On the other hand, several terrestrial chelonian lineages have fully functional (i.e. secretory) and in some cases hyper-developed MGs. Adaptation to terrestrial life coerces changes in the structure and composition of secretory organs, as well as chemosensory receptors^32,52–54^. A quantitative comparison of MG secretions, as well as olfactory receptor diversity between aquatic and terrestrial turtle species, is warranted. Our findings contrast with those for lizards, in which the number of epidermal glands involved in chemosignal production is associated with certain environmental factors such as substrate. Shrub-climbing and arboreal species show a reduction in the number of femoral glands in distinct taxonomic groups of lizards^27,28^. These results have been interpreted as reduced investment into chemical signaling in arboreal species inhabiting environments in which it is less efficient. However, the number of femoral or precloacal pores is not related to climatic conditions (e.g. temperature) in lizards, suggesting strong phylogenetic inertia on this trait^27,29^.

One drawback to our denotation of macrohabitat involves the lumping of several disparate environments under the same macrohabitat category. For instance, there is a striking difference in the terrestrial habitat (arid to semi-arid vs. moist forest) occupied by *Gopherus* and *Manouria*, respectively, both of which harbor MGs. The relatively xeric conditions inhabited by *Gopherus* spp. could have imposed strong selective pressure on chemosignaling, resulting in the evolution of large glands releasing copious amounts of secretions able to persist under extremely hot habitats with rapid signal fade out^8,55^. In contrast, MGs are always smaller and simpler in *Manouria*, the sister group of *Gopherus* inhabiting moist forests of Southeast Asia (compare Fig. 2 and Supplementary Fig. 2). A finer partitioning of habitat categories or MG size/complexity could have provided a more nuanced understanding of the influence of environment on MG expression but would have also lowered sample size and thus, power of the analyses.

### Chemosignaling through MGs and alternative communication channels in turtles

Behavioral experiments on both terrestrial and freshwater turtles have shown the importance of chemical signals during mate choice and intraspecific competition^41,56–58^. The secretions released by MGs have been suggested to play an important role during social interactions in *Gopherus* tortoises^40–42,59^. During courtship, these species perform head bobbing, used as a visual display to other conspecifics, but that may also serve to disperse chemicals from MG secretions during sexual encounters^60^. Head bobbing as well as other head movements displayed during courtship are widespread in chelonians^61–63^, including both species with and without MGs that are phylogenetically distant. This would argue against courtship head movements mediating chemical signaling as a primary function. This behavior might be mainly used as a visual and/or tactile signal, while chemical dispersion through head movements could have been secondarily co-opted in some chelonian taxa such as *Gopherus*. Although distinct types of head movements have been described for several species of turtles (see references^62,64^ for a review), detailed descriptions of courtship are lacking for the vast majority of species. We think that the hypothesis of head movements being co-opted as a way to disperse chemosignals from MGs should be tested in a macroecological approach after compiling a global dataset on turtle courtship behavior. Unfortunately, the exact mechanism of action of MGs remains unknown, especially for freshwater turtles. A potential hypothesis would be that turtles release secretions passively in the aquatic environment (e.g. ponds or streams), and the chemical signals are detected by conspecifics that can then select or avoid these environments on the basis of the acquired information^56^.

Despite the relevance of MGs for chemical communication, it is important to mention that turtles possess other potential sources of chemosignals such as musk or Rathke glands^34,35^, located in the axillary or inguinal regions. Rathke glands produce holocrine secretions that are rich in proteins, mucosubstances and lipid droplets^34^. The function of Rathke glands is not fully understood. While it has been suggested that Rathke glandular secretions would work primarily as a predator repellent, a role in intra- or inter-specific communication is also likely^33,34^. Interestingly, Rathke glands are more taxonomically widespread than MGs, as the former appear in all recent turtles (including sea turtles and other freshwater turtles not belonging to the superfamily Testudinoidea), with the exception of Testudinidae (“land tortoises”) and some Emydidae species in which they are absent (*Chrysemys* complex) or reduced to axillar glands. Winokur and Legler^39^ suggested a reduction in the integumentary complexity of Emydidae. Although MGs are present in one lineage of testudinids (*Gopherus* + *Manouria*), two other testudinid clades exhibit a reduction in epidermal glands (Fig. 2). In contrast, many geoemydids possess several sources of chemosignals (e.g. MGs and Rathke glands), implying that they strongly rely on chemical cues for communication.

Although chemical signaling is ubiquitous in the animal kingdom, many species use multimodal signals that include distinct channels^41,65,66^. The reduction or loss of a signaling channel may be coupled with the expansion of compensatory communicatory senses^54,67^. Therefore, the loss of MGs in a given lineage could be mitigated by development of other channels of communication. Besides chemical signals, chelonians may also use tactile, auditory and visual cues to communicate^62,68^. Available data on turtle communication is scarce (see^62,64^ for a review), which hinders an understanding of how and if signaling channels could be compensated by one another. Many turtle species possess sexually dichromatic color patches, stripes and dots on their bodies, especially on the head and limbs^69–72^. Body color marks might be seasonally displayed and potentially used in mate choice. For instance, most of the riverine *Batagur* species lack MGs but present prominent seasonal sexually dichromatic coloration^73–75^. In addition, a complex innate courtship behavior, including claw vibrations displayed during mating, is known in several species of *Chrysemys*, *Trachemys*, *Pseudemys* and *Graptemys*^64,76^, in which MGs are absent. Moreover, recent studies have evidenced underwater vocalization in turtles^68,77^ that could be under sexual selection in some species. We speculate that a loss of MGs, indicative of a (partially) compromised chemical signaling path (i.e. a constraint of any type), could have exacerbated the development of other channels of communication, such as ornamental coloration, tactile stimuli and/or vocalizations, thereby compensating the loss of secretory function. However, the opposite situation might also be possible, for instance, the hyperdevelopment of a certain trait (i.e. pronounced seasonal dichromatic coloration in *Batagur* or the complex courtship rituals of *Trachemys* and related species) could speed up the loss of other communication channels or organs such as MGs. More data on communication channels in turtles are needed to test these hypotheses under a macroecological framework.

Multiple factors unrelated with alternative communication channels have been shown to influence epidermal glands and chemical signals in reptiles. For instance, sociality in lizards is associated with the presence of femoral glands indicating an important role of chemosignaling on squamate social grouping^78^. In addition, the composition of chemical signals produced by femoral glands is shaped by diet^79–81^, climate conditions^25,82^ and predation pressure^83^. Although in our study we do not focus on chemical composition of MGs, it is possible that any of these factors could have an influence on MG evolution and, more likely, the secretions produced by MGs. Future studies should focus on MG chemistry to clarify how other factors (e.g. climate conditions or diet) may affect chemical signaling.

## Conclusions

Mental glands appear for the first time in the ancestor of the superfamily Testudinoidea and have been lost and/or reduced in multiple instances. Independent gains of MGs were not inferred, implying that MGs are homologous in all chelonians in which they occur. Anatomical and histological examinations further support this finding. In some clades, MGs are evolutionarily plastic with closely related species showing fully developed and likely functional glands, while others exhibit rudimentary forms of the organ. We suggest a scenario in which MGs appeared in an aquatic ancestor and expanded in several clades of aquatic and terrestrial turtles. Although MGs could have first evolved to communicate in water, some land-dwelling chelonian lineages have maintained or even enhanced MG functionality. Under a coarse binary definition, aquatic and terrestrial environments per se do not seem to influence loss of MGs. However, we note that extant species exhibiting marked seasonal dichromatic coloration or complicated courtship typically lack MGs. We suggest that further macroevolutionary studies on chemosignal composition and transmission could clarify the evolution of chemical communication in chelonians.

## Material and methods

### Assessment of mental gland status

In total, we surveyed approximately 700 chelonian specimens for MG status, mostly from museums and live collections (see Table S1; main institutions and sources of specimens are listed in Supplementary Material). Living specimens from private collections and zoos were checked carefully for the presence of MGs with the permission of the owners or responsible persons and following standard rules of animal welfare.

In museum specimens, the chin was stretched and carefully examined for the presence of MGs. In some specimens, MGs are present as swollen and prominent bulges on the underside of the head, in the anterior skin of the throat with openings (orifices) that can be detected after careful inspection. In others MGs resemble small keratinized invaginations^39^, which can readily be discerned using a dissecting microscope. MG occurrence is usually polymorphic within a species (Table S1), typically being more evident in males, but sometimes MGs may also be found in females and juvenile specimens. We therefore examined males, females, juveniles as well as unsexed specimens.

To compile our dataset on MG presence/absence in chelonians, we merged our own assessment with the findings of Winokur and Legler^39^. In Winokur and Legler^39^ all families of recent chelonians were assessed but MGs were only found in four, all belonging to superfamily Testudinoidea (Geoemydidae, Testudinidae, Emydidae and Platysternidae). In another study^84^, no evidence of MGs was found in two species of Chelidae. Therefore, we focused on the families Platysternidae, Emydidae, Geoemydidae and Testudinidae and only assessed one kinosternid species. We considered MGs as present in a species, if these were encountered in at least one individual in our or previous^39^ surveys. Typically, the assessment of MG status in particular species was equivalent in both surveys^39^, differing in only a few species: our position on these controversial cases is summarized in the Supplementary Material (*Controversial taxa and intraspecific variation*). As MGs were never observed in a total of 418 specimens in any other turtle family outside Testudinoidea^39^, we scored MG status in all species from these families as absent (see Supplementary Table S1). Some species of Emydidae and Testudinidae were not checked here, but these were scored as lacking MGs based on our or previous^39^ assessments of closely related taxa (see Table S1). MG status for six geoemydid species for which specimens were unavailable were scored as unknown.

A few species assessed for the status of MGs (see Table S1) were not present in the phylogenetic timetree of Pereira et al.^43^ that was used to assemble the final dataset for main analysis (see below). Therefore, these species were not included in the main analyses. These data were useful for clarifying MG presence in some clades (e.g. *Malayemys*) and therefore are shown in the Supplementary Material. In addition, some of these taxa (e.g. *Cyclemys pulchristiata*) were present in the timetree of Thomson et al.^46^, that was used to run an extra analysis (see below).

### Histological methods

Histological examination was done in 12 dead specimens donated by private breeders. Upon acquisition, all were fixed and stored in 70% ethanol. The turtles died from natural reasons and were not sacrificed for the present study and, thus, no permissions or experimental protocols from institutional committees were required. Specimens from the following species were examined histologically (number of specimens in brackets): *Cuora amboinensis* (5), *C. mouhotii* (1), *Mauremys annamensis* (1), *M. japonica* (1), *M. reevesii* (2) and *Sacalia quadriocellata* (2)*.* Mental glands were excised postmortem and processed for histology using Harris's Haematoxylin and Eosin staining, Periodic Acid-Schiff (PAS) stain and Mallory’s Trichrome according to Ibáñez et al.^37^. In the case of *M. reevesii*, one of the two specimens examined histologically had no clear macroscopic evidence of MGs, in this case the skin of the throat in which MGs are typically located was dissected and processed similarly.

### Inference of ancestral states

We reconstructed ancestral states for two traits, MG status and macrohabitat, using the timetree of Pereira et al.^43^, based on multilocus DNA sequence data for 292 extant turtle species. The major nodes of this phylogeny are well-supported and concordant with other studies using phylogenomic datasets but sparser taxon sampling^49,85^. MG status was coded as present, absent or missing if no information was available as identified for a few species of geoemydids. Very small and reduced (“vestigal”) and very likely functionless glands were coded as present as these represent gradual variation of this trait. Macrohabitat was coded as aquatic or terrestrial. Most turtles are associated with aquatic habitats and only three families of extant chelonians—Emydidae, Geoemydidae and Testudinidae—have true terrestrial species^51,86,87^. While all testudinids are terrestrial, several species of geoemydids and emydids represent fully terrestrial forms as well. However, many others have intermediate lifestyles with different degrees of dependence on aquatic and terrestrial habitats^86–88^. As we used a binary classification, geoemydid and emydid species with a tendency toward terrestriality were coded as terrestrial (see Table S1). All remaining turtle species, including taxa not belonging to these three families with semiaquatic lifestyles (such as for example some side-necked and kinosternid species), were coded as aquatic (Table S1). We inferred ancestral states for MG and macrohabitat using the corHMM function in the corHMM v2.5 R package^89,90^. This function calculates the maximum likelihood estimates of transition rates between states and then uses these values for determining state probabilities for internal nodes of the tree, and can also incorporate “hidden” rate changes across a phylogeny^91^. We constructed four models by modifying the transition (Q) matrix. The first considered all transition rates as equal (ER model). The second allowed for transition rates between states to differ (ARD model). In a third model built only for the MG dataset, we fixed the most recent common ancestor (mrca) of Testudinoidea to gland absent, which effectively enforced independent origins (IO model) of MGs in the two main clades of turtles in which they occur (Emydidae + Platysternidae vs. Testudinidae + Geoemydidae).

The three models described above assume that the process generating the different states at the tips and ancestral nodes is homogenous across all branches of a phylogenetic tree, which may be a major simplification of biological reality. The generalized hidden Markov model^90,91^ implemented in corHMM v2.5 relaxes this assumption by allowing more than one process to affect trait evolution across a phylogeny. This is achieved by constructing > 1 rate categories (i.e. transition matrices) and allowing them to vary across the tree by parameterizing the transitions among rate categories. We constructed three different models, each with two rate matrices (R1 and R2, rate.cat = 2). We specified a model with two equal rates (ER/ER) matrices, another with two all rates different (ARD/ARD) matrices, and finally a mixed ER/ARD model. In all cases, rate category transitions (R1—> R2 and R2—> R1) were allowed to differ.

The same set of models (with the exception of model IO exclusive to the MG dataset) was constructed for both of the traits, MG status and macrohabitat, in separate analyses. In the former, we fixed the root state value to MG absent, while in the latter to aquatic habitat. Akaike’s information criterion corrected for small sample size^92^ was calculated from the log-likelihoods to compare the fit of the models. Each model was run 100 times (nstarts = 100).

### Correlating gland presence and habitat

Next, we asked whether gland status and macrohabitat are correlated in turtles. We used the corHMM function in the corHMM v2.5 package to fit different models of evolution for the two categorical traits^50,90,93^. For two binary characters (X, Y) with states 0 and 1, a maximum of 8 different transition parameters can be defined (disallowing for simultaneous changes in both variables), in a continuous-time Markov chain. Our simplest model set all transition rates equal (ER model) and thus contained only one parameter. We also fitted a model of independent evolution between traits (IND model, 4 parameters) by holding the state of one trait constant while allowing the other to vary (e.g.: 1, 0—> 1, 1) for each of the four possible combinations of states of the two traits. Next, we fitted a model of correlated evolution between traits (ARD model), in which each transition rate between states was allowed to be different, giving a total of 8 free parameters. We also asked whether gland loss depends upon a change in macrohabitat (ARD_loss). To achieve this, we set up a model in which the transition between an aquatic turtle with a gland (0, 1) to an aquatic turtle lacking a gland (0, 0) was set equal to the rate of the transition between a terrestrial turtle with a gland (1, 1) going to a terrestrial turtle without a gland (1, 0). The fifth model (ER/ER) applied the generalized hidden Markov process and consisted of two transition matrices (rate.cat = 2), R1 and R2. Both rate categories were constrained to have equal rates within each matrix, while transitions in rate categories were allowed to differ. The sixth and final model (ER/ARD) included one ER and one ARD matrix, with transition rates between the two matrices set equal, leading to 10 estimated parameters. We refrained from constructing more complex models due to the concomitant increase in parameter space. We fixed the root state value to MG absent and aquatic habitat, and for each model specified 100 maximum likelihood analyses with different initial parameters (nstarts = 100).

Because the best models in our set consistently revealed differences in the evolutionary process underlying trait evolution in Testudinoidea compared to other chelonians (see Results and Fig. 5), we ran a subset of the models (ER, IND, ARD, ARD_loss) after pruning the tree to only include this clade (151 terminal taxa).

While this study was under review, a new chelonian molecular phylogeny was published^46^. Although this new phylogenetic hypothesis recapitulates most relationships among major turtle clades from previous studies^43,85^, it shows some differences in the arrangement of taxa within turtle families, e.g. in Geoemydidae. To test whether these phylogenetic rearrangements influence our results, we used the maximum clade credibility tree from the divergence time analysis of Thomson et al.^46^, pruned to include only species in our MG dataset, in a separate set of analyses aimed at inferring and correlating ancestral states in MGs and macrohabitat. Models for each trait were run in corHMM as described above. The dataset used for this analysis includes less species than the dataset assembled in Pereira et al.^43^. However, in this analysis we included taxa that possess MGs but that were not present in Pereira et al.^43^, such as *Cyclemys pulchristriata* (assessed in our survey, see Table S1) or *Gopherus morafkai* (MGs are reported previously as in other *Gopherus*^94,95^). Also, a few species not present in Pereira et al.^43^ from families and genera typically lacking MGs (see above and Table 1) were included when present in Thomson et al.^46^.

## Supplementary Information

Supplementary Information 1.

Supplementary Information 2.

Supplementary Information 3.

## Data Availability

Generated datasets, input files and code for the main analysis—using Pereira et al., (2017)^43^ timetree—are available in the supplementary material and Figshare (http://doi.org/10.6084/m9.figshare.14258585).
