# Supplementary material for: Evolutionary history of mental glands in turtles reveals a single origin in an aquatic ancestor and recurrent losses independent of macrohabitat
Source: Sci Rep. 2021 May 17;11:10396. doi: 10.1038/s41598-021-89520-w (PMC8129087; doi:10.1038/s41598-021-89520-w)
Supplement: Supplementary file 1 — Supplementary Information 1. [file 41598_2021_89520_MOESM1_ESM.pdf]

Evolutionary history of mental glands in turtles reveals a single origin in an aquatic ancestor and recurrent losses independent of macrohabitat

Supplementary Material

Alejandro Ibáñez, Uwe Fritz, Markus Auer, Albert Martínez-Silvestre, Peter Praschag, Emilia Załugowicz, Dagmara Podkowa, Maciej Pabijan

Supplementary figures and captions:

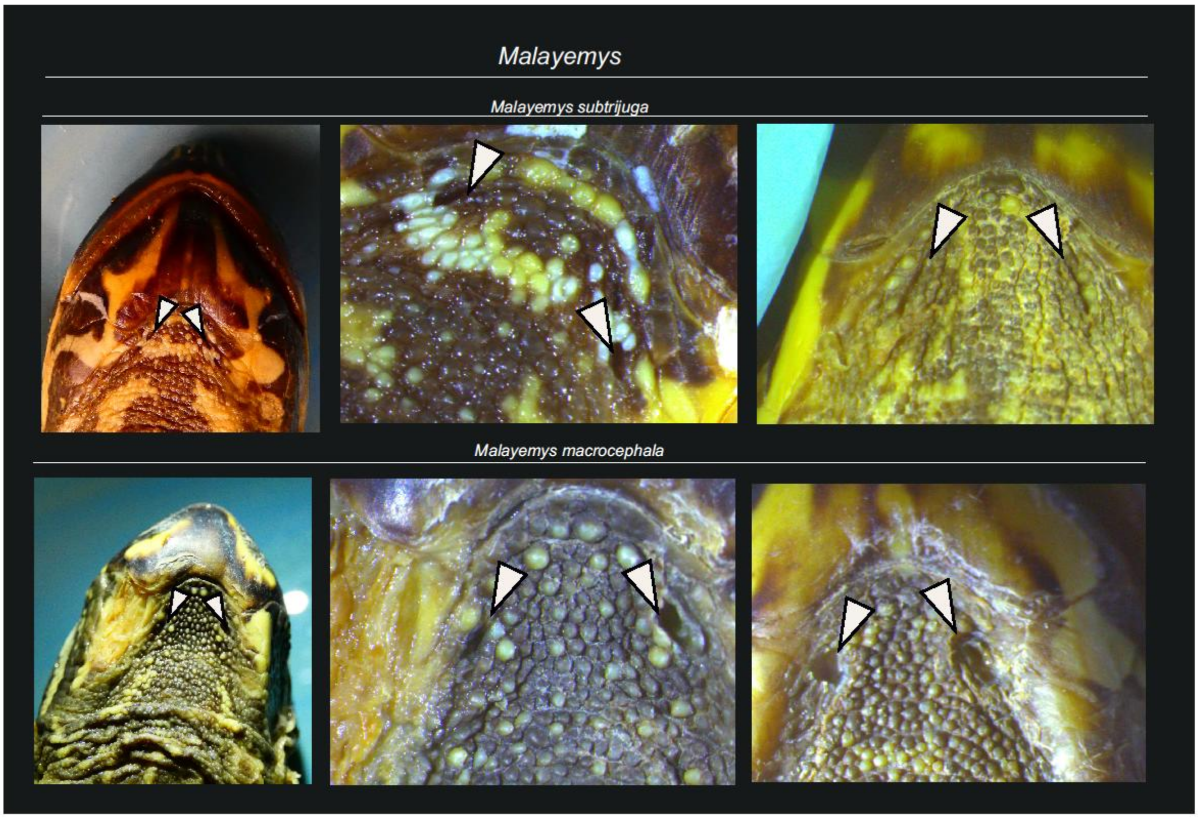

Supplementary Figure S1. Macroscopic aspect of MGs in some specimens of *Malayemys subtrijuga* (collection numbers for the specimens pictured: MTD 22525; MTD 26087) and *Malayemys macrocephala* (MTD 28195 and MTD 28196). Arrowheads indicate the approximate location of the MG orifices.

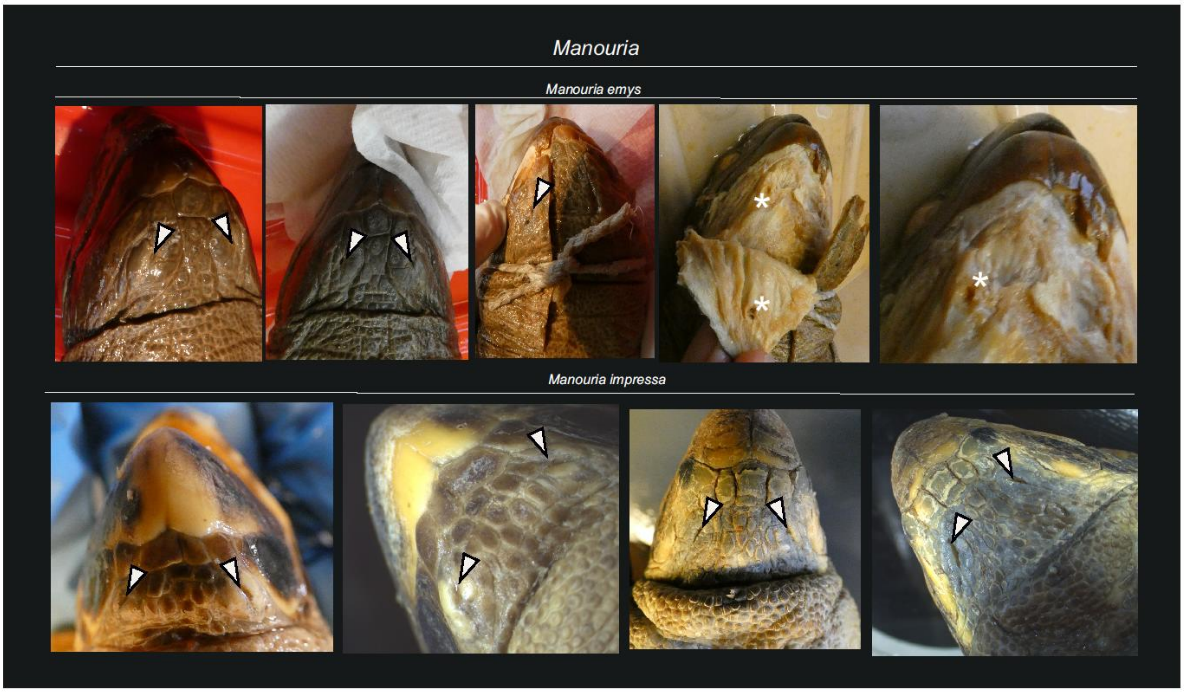

Supplementary Figure S2. Macroscopic aspect of MGs in some specimens of *Manouria emys* (SMF 70492 and SMF 22345) and *Manouria impressa* (MTD 40718 and MTD 41343). Arrowheads indicate the approximate location of the MG orifices. Asterisks are used to highlight the inner part of MGs in a specimen with detached skin of the neck.

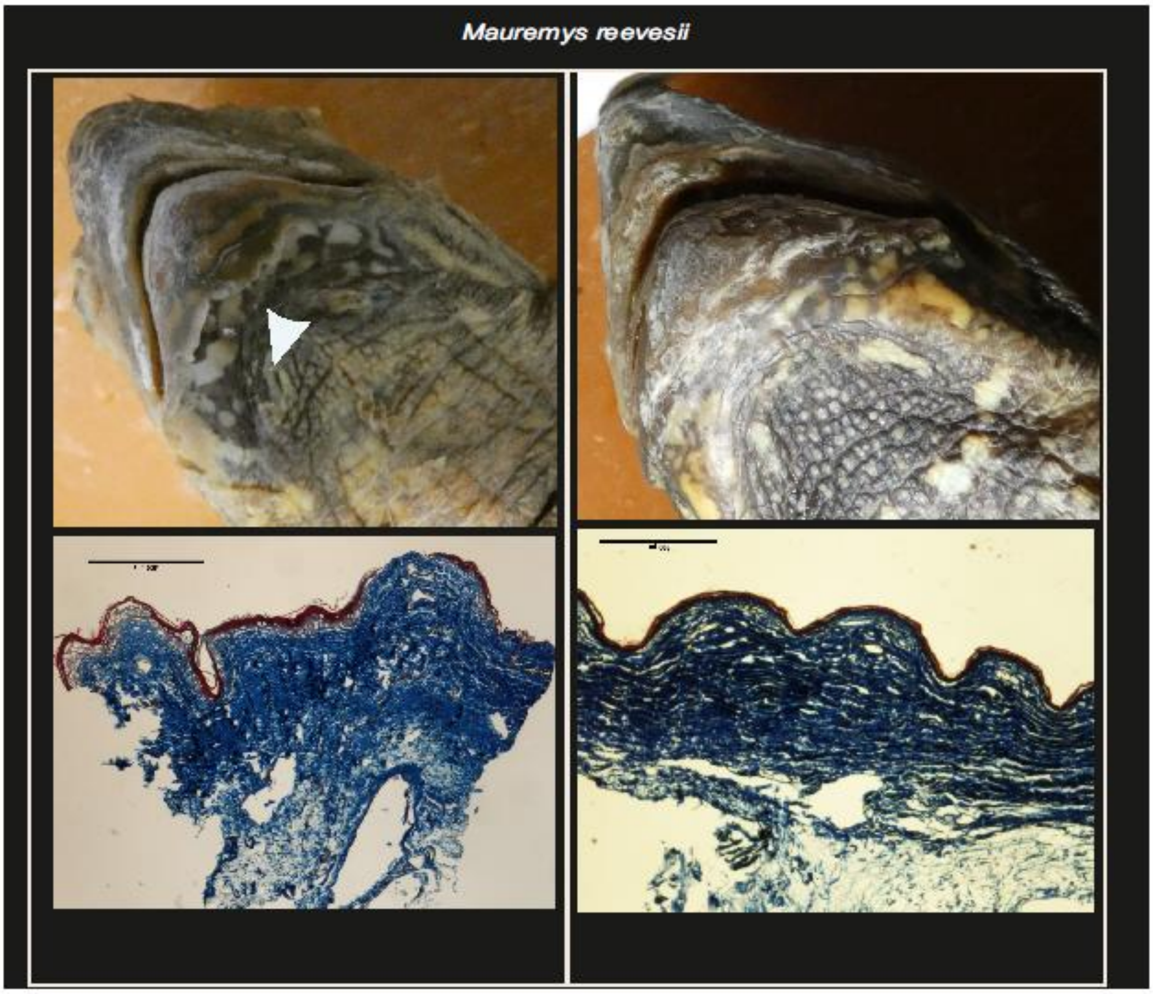

Supplementary Figure S3. Macroscopic (top) and histological (bottom) aspect of two females of *Mauremys reevesii*. The specimen on the left has a very reduced (“vestigal”) form of MGs (arrowhead pointing to the small invagination). Pictures on the right side are from another specimen without any sign of MGs (i.e. absence of MGs). The staining for the histological preparation shown is Mallory’s Trichrome.

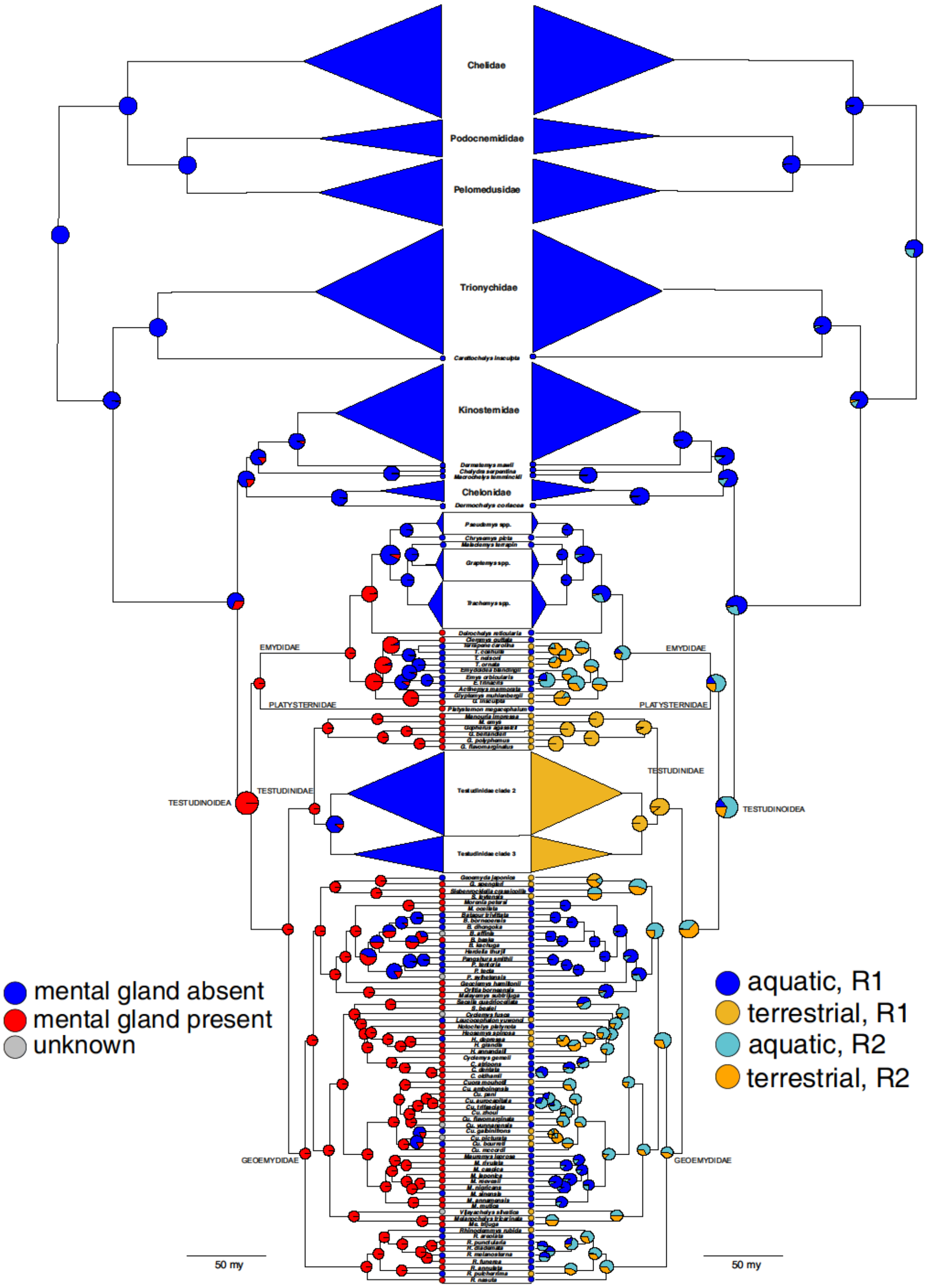

Supplementary Figure S4. Maximum likelihood inference of ancestral states in mental gland (MG) status (left) and macrohabitat (right) in extant Testudinoidea based on the molecular timetree of Pereira et al. (2017)[1]. Pie charts denote likelihoods under the all rates different (ARD) model for MG status. The generalized hidden Markov process with two equal rate (ER) transition matrices (R1, R2) was used to model the evolutionary history of macrohabitat. Circles at tips show MG and macrohabitat status for extant species. Species names at the terminal labels as in the original publication[1], inclusive of putative misidentifications.

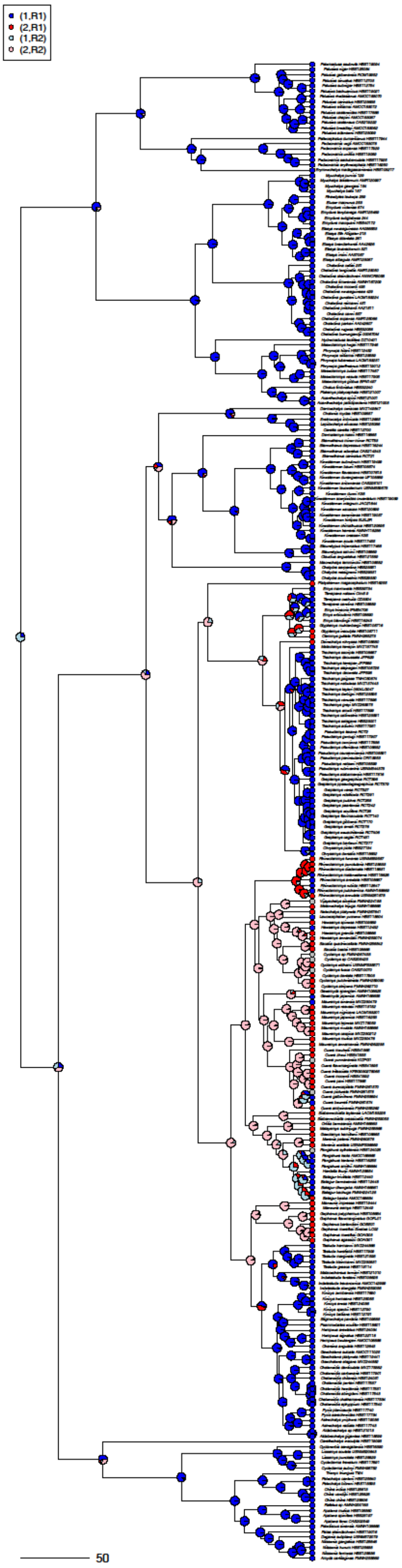

Supplementary Figure S5. Maximum likelihood inference of ancestral states in mental gland (MG) status in extant chelonians based on the molecular timetree of Thomson et al. (2021)[2], using a generalized hidden Markov model allowing for two different transition matrices to account for transitions between states across the tree (ARD/ARD model). Pie charts show marginal likelihood estimates for states at ancestral nodes. Blue colors denote MG absence (for matrices R1 and R2), while red and pink denote MG presence (for matrices R1 and R2). Species names and alphanumeric codes at the terminal labels as in the input datasets from the original publication (found in Dryad repository)[2], inclusive of putative misidentifications.

List of sources for specimens checked for MG presence/absence in our study:

Museum für Tierkunde, Senckenberg Dresden, Dresden, Germany (MTD)
Forschungsinstitut und Naturmuseum Senckenberg, Frankfurt am Main, Germany (SMF)
Turtle Island, Graz, Austria
Wroclaw Zoo, Wroclaw, Poland
Plock Zoo, Plock, Poland
Dresden Zoo, Dresden, Germany
Catalonian Reptile and Amphibian Rehabilitation Center (CRARC), Masquefa, Spain
Private collections/breeders

Controversial taxa and intraspecific variation

In several species we obtained results that differed from those of Winokur and Legler (1975)[3]. Our gross assessment of an adult male and two other specimens (juveniles) of *Batagur baska* showed no evidence of MGs. However, MGs were present in the single individual of *B. baska* assessed previously[3]. Since the publication of Winokur and Legler (1975)[3], it was discovered that *B. baska* sensu lato consists of two genetically and morphologically differentiated species, *B. baska* sensu stricto and *B. affinis* that were previously lumped together[4]. While we lack information on the precise origin of the individual checked previously in Winokur and Legler (1975)[3], it is stated that it comes from a zoo in Malaysia. Therefore, it seems likely that this individual was indeed *B. affinis* instead of *B. baska*, given that only the former species occurs in Malaysia. If this would be the case, then MGs should be present in *B. affinis*. However, this scenario would have no (or very little) effect on our results (e.g. number of MG losses), as it would leave the status for *B. baska* as uncertain (or potentially absent). Given this controversy and considering that sample size for the rare and highly endangered species *B. baska* and *B. affinis* is small, we designated *B. baska* as having MGs while leaving *B. affinis* as unknown for MG status. However, we also consider another possibility in which MGs are absent in *B. baska* and "unknown" in *B. affinis*. This would decrease the number of MG losses by three, bringing the total minimum number of independent losses of MGs down from 18 to 15.

Mental glands were also reported previously[1] for *Geoemyda spengleri* (present in 2 out of 6 specimens), *Heosemys spinosa* (2 out of 3 specimens), *Cuora flavomarginata* (3 out of 10 specimens) and *Rhinoclemmys funerea* (5 out of 21 specimens). Our macroscopic assessment of *G. spengleri* (3 specimens), *H. spinosa* (1), *C. flavomarginata* (12) and *R. funerea* (4) did

not find any evidence of MGs in these species. Nonetheless, all of these species were designated as having MGs according to the previous results[3].

We found MGs in two genera, *Malayemys* and *Manouria* (Figs. S1-S2), for which glands were not reported by Winokur and Legler (1975)[3]. In *Malayemys subtrijuga* a total of 16 specimens were checked previously, including one specimen for histology, without any sign for the presence of MGs[3]. Our macroscopic assessment of 6 specimens of *M. subtrijuga* showed that two had small invaginations that resemble a reduced form of MGs. These two specimens were sexed as females, while the four males assessed showed no signs of MGs. The high polymorphism of MGs, together with a relatively low sample size, could explain that MGs were found only in females in *M. subtrijuga* (and potentially other species). We also checked some specimens of *M. macrocephala*, a closely related species that was not included in the phylogenetic tree used in our study. Our gross assessment of specimens of *M. macrocephala* revealed the presence of invaginations that resemble reduced forms of MGs. These were slightly more developed than *M. subtrijuga*, providing further evidence of MG presence in this genus (Fig. S1). According to Winokur and Legler (1975)[3], *Gopherus* is the only genus among testudinids having MGs, but they did not check *Manouria*, the sister group of *Gopherus*. A more recent study based on living and museum specimens of *Manouria* failed to find MGs[5]. Our macroscopic assessment of *Manouria emys* and *M. impressa* revealed the existence of MGs in this genus (Fig S2). MGs were obvious just in a few specimens and reduced and even absent in other specimens, which could therefore explain why these glands were not found before[5]. It is important to emphasize that MGs in *Manouria* are much smaller than those of *Gopherus*, but nonetheless quite obvious in some specimens (see Fig. S2).

The case of the genus *Rhinoclemmys* deserves special mention due to difficulties in unambiguously assigning MG status to some species. In *R. punctularia*, Winokur and Legler (1975)[3] did not find MGs in a total of 36 specimens checked, i.e. in a relatively large sample size. In our case 11 specimens were grossly assessed and several showed reduced and cryptic MGs, slightly more evident in one of the live adult males checked (Fig. 2). Therefore, although MGs are reduced in *R. punctularia*, the macroscopic aspect of this individual showed a better developed MG than in any other specimen of *Rhinoclemmys* examined in our study, and potential functionality of MGs in this species cannot be ruled out. Another study focusing on the courtship behavior of *R. pulcherrima incisa* reported the presence of MGs as a personal observation of the author, but unfortunately no information on the polymorphism, macroscopic aspect or variation among individuals is available[6]. Summing up our survey and

the previous study by Winokur and Legler (1975)[3], a total of 40 specimens, including a few specimens of the subspecies *R. p incisa* checked herein, were macroscopically assessed, but no evidence of MGs was found. Therefore, we consider *R. pulcherrima* as lacking MGs. In the case of *R. annulata*, our gross assessment of 11 specimens showed evidence of inconspicuous MGs in four specimens, but the glands were extremely reduced (vestigial, but most of the individuals assessed were unsexed). However, in Winokur and Legler (1975)[3], a histological preparation of a mental gland of *R. annulata* showed epidermal vacuolated cells indicating that glands could be actively secreting and therefore it is possible that in some adult males MGs could be potentially functional.

Mental glands are enlarged and more prominent during the reproductive season, and are usually larger and more complex in males than in females or juveniles. Our assessment of MGs was based on specimens mostly from museum collections, for which information on the breeding status of the individuals was not available. Furthermore, in many instances we had a limited number of specimens available, as turtles and tortoises are typically uncommon. This group includes many endangered species, with more than half of the chelonian species threatened with extinction[7]. This situation renders it hard to obtain material for many species. Thus, seasonal and inter-individual variation in the expression of MGs may have obscured MG status in some species, e.g. those described as having reduced MGs may develop larger glands during the breeding season. Yet, our dataset encompasses an unprecedented large number of specimens that were carefully checked for MGs, making it reliable for the majority of chelonian species. Any future change in MG status should be limited to a few species with low sample sizes (Table S1). Therefore, we conclude that ancestral state inference and habitat correlations would be little influenced by this issue.

Details on the structure and evolution of MGs in turtles

Although the general anatomical organization of MGs is preserved across species, macroscopic and histological structure of the glands varies across taxa and such variation is likely gradual[1] (and results of the present study). MGs exhibit sexual dimorphism; in general they are less developed in females than in males. Our histological examination of a subset of specimens corroborated a simpler histological structure in females being in some cases extremely small in size. A lack of holocrine secretions in the lumen of the gland in females could indicate either a rudimentary condition or seasonal inactivity. In some species of

testudinoids, MGs – when present – were always very reduced in size and are very likely vestigial. Despite being extremely small, "vestigial" MGs can be distinguished from folds present in the chin because these are well-defined invaginations close to the jaw where MGs occur (see Fig. S3 for a comparison between one specimen with "vestigial" MGs and another lacking MGs). In any case, the structure and morphology of very reduced forms of MGs argues against their functionality.

Evidence for well-developed and prominent MGs was found in a few testudinoid species (especially in *Gopherus*, family Testudinidae, and in the geoemydid genera *Siebenrockiella*, *Mauremys* and *Cuora*). Less obvious, but still noticeable, MGs were present in other species (Geoemydidae: *Cyclemys*, *Melanochelys*, *Morenia*, *Sacalia*, *Orlitia*; Testudinidae: *Manouria*; Emydidae: *Deirochelys*; Platysternidae: *Platysternon*). This indicates that functional glands may be present in several groups. According to the 292 species included in our analysis (Table S1), MGs are most prevalent in Geoemydidae (present in 42 species), but this family also shows large variation in the development of this trait. For instance, *Siebenrockiella crassicollis*, *Cuora amboinensis* and *Mauremys leprosa*, all aquatic species, exhibited obvious and quite prominent MG structures (especially in *S. crassicollis*, see Fig. 2 for the macroscopic aspect of MGs in *S. crassicollis*) that produce secretions[3,8]. However, MGs were reduced in other closely related species (e.g. *M. reevesii*) or even lost (e.g. *M. sinensis*; clade *Cuora picturata* + *C. bourreti* + *C. galbinifrons*). Among emydids, the glands are generally lost or extremely reduced (*Clemmys guttata* and *Glyptemys insculpta*), with the exception of *Deirochelys reticularia*, in which MGs are clearly more developed than in the rest of the Emydidae. We agree with Winokur and Legler (1975)[3] that some taxa, such as *Glyptemys insculpta*, *Clemmys guttata* and several geoemydids, such as *Mauremys reevesii* and *Geoemyda spengleri*, have cryptic and likely nonfunctional MGs (however, we did not find MGs in *G. spengleri*, including one live male). Very reduced or vestigial MG forms were also found in *Malayemys subtrijuga* and *Heosemys grandis* – but in these two cases we checked only a few specimens. Our assessment of gross morphology suggests that several species have vestiges of the glands that resemble small invaginations of the skin and reinforce the idea of non-functional MGs for many of these taxa.

The substantial plasticity of MG development within and between species indicates that the previous classification of these glands in two categories proposed by Winokur and Legler (1975)[3] is artificial. According to this classification, there are two categories of MGs: Class I (highly prominent and well-developed multilobed glands) and Class II (these range from

saccular glands with holocrine secretions to highly keratinized invaginations; likely rudiments). Class I glands were suggested to be restricted to the genus *Gopherus,* while Class II glands were inferred to be present in other species (families Platysternidae, Emydidae and Geoemydidae). However, the intrinsic gradual variation in MG expression across species shows the limitations of using this classification.

References

1 Pereira, A. G., Sterli, J., Moreira, F. R. & Schrago, C. G. Multilocus phylogeny and statistical biogeography clarify the evolutionary history of major lineages of turtles. *Molecular Phylogenetics and Evolution* **113**, 59-66 (2017).

2 Thomson, R. C., Spinks P. Q. & Shaffer, H. B. A global phylogeny of turtles reveals a burst of climate-associated diversification on continental margins. *Proceedings of the National Academy of Sciences* **118**, e2012215118 (2021).

3 Winokur, R. M. & Legler, J. M. Chelonian mental glands. *Journal of Morphology* **147**, 275-291 (1975).

4 Praschag, P., Sommer, R. S., McCarthy, C. & Gemel, R. Naming one of the world's rarest chelonians, the southern *Batagur*. *Zootaxa* **1758**, 61–68.

5 Le, M., Raxworthy, C. J., McCord, W. P. & Mertz, L. A molecular phylogeny of tortoises (Testudines: Testudinidae) based on mitochondrial and nuclear genes. *Molecular Phylogenetics and Evolution* **40**, 517-531 (2006).

6 Hidalgo, H. Courtship and mating behavior in *Rhinoclemmys pulcherrima incisa* (Testudines: Emydidae: Batagurinae). *Transactions of the Kansas Academy of Science* **1903**, 82-95 (1982).

7 Stanford, C. B. *et al.* Turtles and tortoises are in trouble. *Current Biology* **30**, R721-R735 (2020).

8 Ibáñez, A. *et al.* The chemistry and histology of sexually dimorphic mental glands in the freshwater turtle, *Mauremys leprosa*. *PeerJ* **8**, e9047 (2020).
